# Distinct nitrogen isotopic compositions of healthy and cancerous tissue in mice brain and head&neck micro-biopsies

**DOI:** 10.1186/s12885-021-08489-x

**Published:** 2021-07-13

**Authors:** M. Straub, D. M. Sigman, A. Auderset, J. Ollivier, B. Petit, B. Hinnenberg, F. Rubach, S. Oleynik, M.-C. Vozenin, A. Martínez-García

**Affiliations:** 1 Institute of Radiation Physics, Lausanne University Hospital and University of Lausanne, 1007 Lausanne, Switzerland; 2 Max Planck Institute for Chemistry, 55128 Mainz, Germany; 3 Department of Geosciences, Princeton University, Princeton, NJ 08544 USA; 4 Radiation Oncology Laboratory/DO/Radio-Oncology/CHUV, Lausanne University Hospital and University of Lausanne, 1011 Lausanne, Switzerland

**Keywords:** Micro-biopsies, Diagnostics, Cell metabolism, Nitrogen isotopes

## Abstract

**Background:**

Cancerous cells can recycle metabolic ammonium for their growth. As this ammonium has a low nitrogen isotope ratio (^15^N/^14^N), its recycling may cause cancer tissue to have lower ^15^N/^14^N than surrounding healthy tissue. We investigated whether, within a given tissue type in individual mice, tumoral and healthy tissues could be distinguished based on their ^15^N/^14^N.

**Methods:**

Micro-biopsies of murine tumors and adjacent tissues were analyzed for ^15^N/^14^N using novel high-sensitivity methods. Isotopic analysis was pursued in Nude and C57BL/6 mice models with mature orthotopic brain and head&neck tumors generated by implantation of H454 and MEERL95 murine cells, respectively.

**Results:**

In the 7 mice analyzed, the brain tumors had distinctly lower ^15^N/^14^N than healthy neural tissue. In the 5 mice with head&neck tumors, the difference was smaller and more variable. This was at least partly due to infiltration of healthy head&neck tissue by tumor cells. However, it may also indicate that the ^15^N/^14^N difference between tumoral and healthy tissue depends on the nitrogen metabolism of the healthy organ in question.

**Conclusions:**

The findings, coupled with the high sensitivity of the ^15^N/^14^N measurement method used here, suggest a new approach for micro-biopsy-based diagnosis of malignancy as well as an avenue for investigation of cancer metabolism.

**Supplementary Information:**

The online version contains supplementary material available at 10.1186/s12885-021-08489-x.

## Background

The stable isotopes of biologically important elements are widely used in biomedical research and medical applications [1–7]. The stable isotopes of carbon, hydrogen, oxygen, sulfur and nitrogen have traditionally been the focus [5, 8–11]. More recently, the list of elements amenable to isotopic analysis has expanded greatly, e.g., to various metals [6].

In stable isotope studies, there are two complementary approaches. The first approach is isotope labelling [7, 12]. This approach starts with a substrate that is enriched in the rare isotope of a given element. The rare isotope-labelled substrate is introduced to a biological or biochemical system, and isotope ratio measurements then determine the fate of the labelled element and the rates of the processes transforming it from one chemical form to another or transporting it from one site to another. The acquired information is relatively direct. Moreover, discrimination among the isotopes is most often a negligible factor. As a result, interpretation of the data is relatively straightforward. However, the approach also has a range of limitations. In particular, these short-term experiments may not accurately reflect the long-term average rates of processes. In the context of medical diagnosis, there is also the need to administer an appropriate substrate with the isotope label; this is not always possible.

The second approach, and the focus of the current study, is natural abundance isotopic analysis. In this approach, the naturally occurring isotopic ratios of different chemical species and/or biological materials are analyzed (e.g., [13–17]). The differences in isotopic ratios among samples are interpreted in terms of the small but measurable isotopic discriminations that occur with most chemical processes. Natural abundance studies are inherently more integrative than isotope labelling approaches. However, interpretation of the data is contingent on prior knowledge of the isotope discriminations associated with the relevant reactions and processes. Moreover, variations in natural abundance isotope ratios are far less pronounced than in isotope labelling studies, requiring extremely precise measurement of the isotope ratios (e.g., [14, 18]).

With the improvement and automation of analysis methods, discussed below, there has been a recent blossoming of natural abundance stable isotope studies in the medical sciences [1, 3, 6]. This work has focused variously on healthy metabolism [19–22], cancer [14–18, 23–27], and other diseases [4, 13]. Of particular interest here is the recent focus on metabolic interactions in the tumor environment [17, 23, 28–33]. This work promises to advance our understanding of cancer metabolism as well as to offer new diagnostic approaches. However, in some cases, such as in stable isotope studies of nitrogen (N), the sample size required for natural abundance isotopic analysis has stood as an impediment (e.g., [14, 17]).

A common feature of cancer cell metabolism is the ability to acquire essential nutrients from a frequently nutrient-poor tumor microenvironment. Several studies have highlighted the importance of N uptake, especially in the form of glutamine, for cancerous cell growth [23, 25, 33, 34]. In fact, the increased N demand observed in cancerous cells has been proposed as a metabolic hallmark of tumor cell metabolism [32]. However, the interplay between nutrient uptake and excretion on a cellular level remains unclear.

For breast cancer cells, Spinelli et al. [33] describe the active recycling of catabolically produced ammonium (NH_4_^+^), normally considered a toxic by-product to be eliminated from the cellular environment. This recycled ammonium helps to satisfy the increased N requirement of rapidly proliferating tumor cells. The re-assimilation of catabolic ammonium is proposed to occur through reductive amination of α-ketoglutarate mediated by the enzyme glutamate dehydrogenase, which is overexpressed (relative to healthy tissue) in different cancer types [33].

With regard to the N isotopes, de- and trans-amination reactions generally result in a series of ^15^N-depleted N by-products (e.g., ammonium, urea, and uric acid), which are eliminated from the cellular environment and ultimately excreted from the organism [22, 35]. As a consequence of this preferential elimination of ^15^N-poor N from the organism, the body tissue of a heterotrophic organism is typically elevated in ^15^N/^14^N with respect to its N source (diet) [20, 36–39]. However, it has been observed that some marine heterotrophic organisms containing photosynthetic symbionts (e.g., shallow water stony corals and some species of planktonic foraminifera) do not express this characteristic (“trophic”) ^15^N enrichment with respect to their diet [40–44]. This observation suggests that the photosynthetic symbionts use the ^15^N-poor ammonium released by the metabolism of the host to synthesize new organic compounds that are kept within the host-symbiont system, potentially offering a competitive advantage in their N-poor surface ocean habitats [40]. This internal recycling of N minimizes the efflux of ^15^N-poor ammonium to the environment and keeps the ^15^N/^14^N of the host organism close to that of its diet.

By analogy with these observations, we hypothesize that the reincorporation of low-^15^N metabolic N in cancer cells results in a measurably lower ^15^N/^14^N of tumors relative to the surrounding heathy tissue, in which low-^15^N metabolic N is not recycled but rather released to the extracellular environment. Such a ^15^N/^14^N difference could provide an independent metric by which to identify and characterize cancerous tissue, as well as to study the N metabolism of different cancer types. On a statistical basis, one study of cancer in human patients has observed a significantly lower ^15^N/^14^N in tumor relative to healthy tissue [18] while other studies have not [15, 17]. For practical reasons and due to the abiding consideration of patient welfare, none of these studies undertook comparisons within individual organisms. More direct comparison of cancerous and healthy tissue as well as greater control in the experimental design would shed light on this question.

In this study, we first introduce a novel analytical method that allows us to perform accurate measurements of the ^15^N/^14^N of tissue micro-biopsies and cancer cell lines with extremely small sample amounts (i.e., 5 nmol N, roughly 2000 cells). This represents a > 100-fold reduction in sample size with respect to previous techniques used for measuring ^15^N/^14^N in tissue samples (e.g., [14, 17]). Second, we use this method to investigate whether, within a given organ of individual mice, malign and benign tissues could be distinguished on the basis of their ^15^N/^14^N. We used well-controlled experimental Nude and C57BL/6 mice models presenting with mature orthotopic brain and head&neck tumors generated by orthotopic implantation of H454 and MEERL95 murine cells, respectively.

## Methods

### Nitrogen isotopic analysis

The method used to measure the δ^15^N of cancerous and healthy tissue biopsies – known as the “persulfate-denitrifier” method – was adapted from previous studies, in which it was used for the analysis of fossil-bound organic material [40, 41, 44–52] as well as for bulk suspended particles and flow-cytometrically sorted cells filtered from ocean waters [53–55]. In this method, the N sample is converted to nitrate in a basic potassium peroxydisulfate solution [56], and nitrate is then converted to nitrous oxide (N_2_O) by denitrifying bacteria that lack an active nitrous oxide reductase enzyme, with N_2_O product undergoing isotope ratio mass spectrometry [57]. The isotopic analyses were performed at the Max Planck Institute for Chemistry, Mainz (Germany). Sample organic N was oxidized to nitrate in a 4 ml combusted glass vial using 1 ml of basic potassium peroxydisulfate solution (1 g potassium peroxydisulfate and 1 g NaOH dissolved in 100 ml high purity water (HPW)). To lower the analytical blank associated with the oxidizing solution, the potassium peroxydisulfate (Sigma Aldrich) was recrystallized three times. The recrystallization, preparation and addition of potassium peroxydisulfate solution was performed in a specially designed clean room equipped with charcoal and particle filters to minimize potential N contamination. The vials were then autoclaved at 120 °C for 65 min on a slow-vent setting (1.5 h including warm-up and cool-down times). Ten 4 ml vials containing only 1 ml of the potassium peroxydisulfate solution were prepared in each batch; and 5 of these vials were combined to estimate the size and ^15^N/^14^N of the procedural blank. International amino acid reference materials, USGS40 [58] and USGS41 [59], were analyzed in triplicate in each batch as an independent monitor of the oxidation and overall precision and accuracy of the method. The nitrate content of each sample was determined after the oxidation step by reduction to nitric oxide using vanadium (III), followed by chemiluminescence detection [60]. The sample nitrate was then bacterially converted to N_2_O and analyzed by isotope ratio mass spectrometry [57, 61] following the updated protocols and instrumentation described in Weigand et al. [62]. Two international nitrate isotope reference materials, IAEA-NO-3 [63] and USGS34 [64], as well as two bacterial blanks were measured in each analysis batch. From here on, ^15^N/^14^N is expressed in terms of δ^15^N, where δ^15^N (in permil, ‰) = ([(^15^N/^14^N)_sample_/(^15^N/^14^N)_air_]-1)*1000. Using the nitrate reference materials IAEA-NO-3 and USGS34, the isotopic analyses were calibrated to yield a δ^15^N value relative to air N_2_, the universal isotopic reference. The oxidized samples were corrected for contribution of the measured blank associated with the oxidation of organic N to nitrate. Experimental data for cell and tissue samples as well as for amino acid reference standards are tabulated in the Additional File, SI Tables 1, 2, 3 and 4.

In addition, the performance of the method in relevant biological materials was tested with breast cancer cells (MCF7, from ATCC) and cervical cancer cells (HeLa, from ATCC) prepared at the Lausanne University Hospital (Switzerland). Cells were grown in vitro as a monolayer culture (75 cm^2^ flasks) in Dulbecco’s Modified Eagle’s Medium (DMEM, GIBCO) with 10% fetal bovine serum (FBS) at 37 °C in an atmosphere of 5% CO_2_. Once the cell sample was confluent, cells were synchronized before harvesting and cell counting. Viability of cells was 95% for MCF7 and 98% for HeLa. After harvesting with Trypsin, the cell samples were rinsed 3 to 4 times with phosphate buffered saline (PBS) to remove any residual N from the medium used. Multiple subsamples of 0.5 million cells were prepared (20 subsamples). At the time of processing for isotopic analysis, cell line dilution series were prepared, with samples of 40000, 20000, 12000, 8000, 4000 and 2000 cells. Between 2 and 6 samples per cell dilution were analyzed.

### Mice models and tissue biopsy protocol

We employed experimental Nude and C57BL/6 mice models presenting with mature orthotropic brain and head&neck tumors generated by orthotopic implantation of H454, Glioblastoma (GBM), and MEERL95 (HPV+ Squamous cell carcinoma) murine cells, respectively. Tissue biopsies were taken directly after sacrificing the mice (cervical dislocation followed by exsanguination to preserve lung physiology). Samples from tumor, adjacent tissue referred to here as “tumor bed” and corresponding healthy tissue (no tumor cell infiltration, e.g., taken in the contralateral region of the brain of the same mouse for the brain tumor model) were taken whenever possible (Additional File, SI Figs. 1 and 2). The samples were rinsed in PBS and then stored at − 80 °C until processing for measurement.

**Fig. 1:**
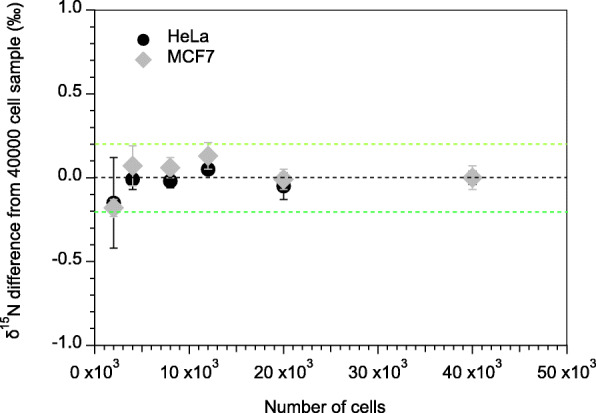
δ^15^N difference of all cell dilutions of MCF7 and HeLa cells relative to the samples with 40,000 cells. Two to six samples per dilution were measured. Error bars indicate standard deviations calculated from 2 to 6 replicates. Green dotted lines indicate ±0.2‰ differences from the average of the analyses of 40,000-cell samples. Average δ^15^N for all MCF7 samples measured is − 1.55‰ ± 0.11‰ vs. air (1 s.d.; *n* = 18), and average δ^15^N for HeLa is − 1.46‰ ± 0.11‰ vs. air (1 s.d.; *n* = 20)

**Fig. 2:**
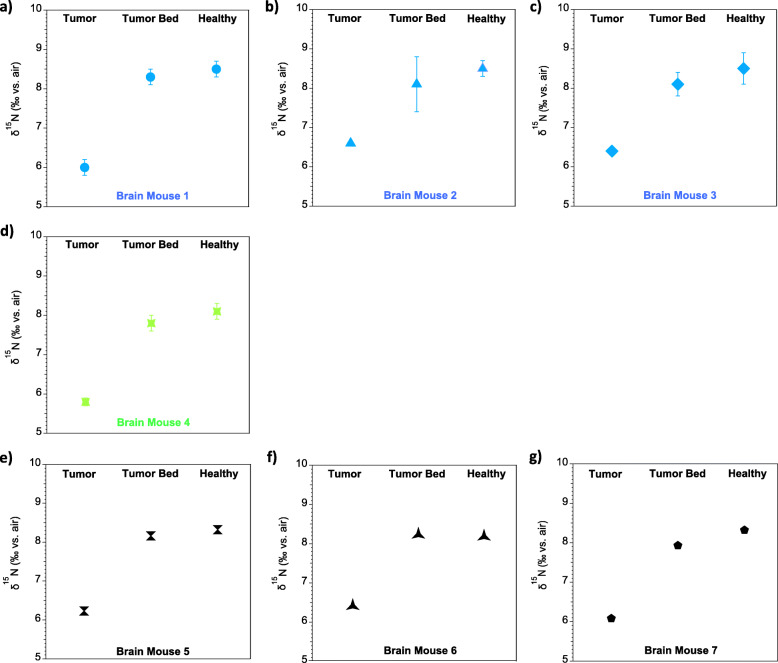
δ^15^N measurements of the mouse brain tumor model. **a-c** Brain tumor biopsies from April 2019, with 2 replicates of each tissue type (tumor, tumor bed, healthy tissue) per mouse and 3 mice measured; **d** Brain tumors from October 2019, with 2 replicates of each tissue type per mouse and 1 mouse measured; **e-g** Brain tumors from November 2019 with 1 sample of each tissue type per mouse and 3 mice measured. Error bars indicate standard deviations of replicates where replicates were measured

Data from brain tumors are reported for three experiments (7 mice in total). The biopsies for these experiments were collected in April 2019 (3 mice, sacrificed 38 days after implantation), October 2019 (1 mouse, sacrificed 46 days after implantation) and November 2019 (3 mice, mouse 5 and 7 sacrificed 35 days after implantation, mouse 6 at 32 days). These samples where measured in two batches in April 2019, and November 2019. The head&neck tumors are reported for two experiments (5 mice in total). The biopsies for these experiments were collected in March 2019 (3 mice, sacrificed 18 days after implantation) and August 2019 (2 mice, sacrificed 19 days after implantation). Isotopic analyses were done in April 2019 and September 2019. Table 1 summarizes the samples per mouse and tissue type taken for each experiment. Throughout the study, sampling and analysis replication were as follows. From each mouse, a sample was taken of a given tissue type (tumor, tumor bed, or healthy tissue). Depending on the sampling campaign, one or multiple subsamples were taken from a given tissue type for isotopic analysis (Table 1). In all cases, the number of subsamples taken was equivalent for tumor and healthy tissue. Each subsample was analyzed only once. Thus, the total number of analyses per tissue type is equivalent to the number of subsamples taken.

**Table 1:** Summary of the sampling scheme

***Time of sampling***	***Number of mice***	***Subsamples per tissue per mouse***
**Brain tumors**		
April 2019	3	2
October 2019	1	2
November 2019	3	1
**H&N tumors**		
March 2019	3	3
August 2019	2	2

### Statistical data analysis

Cell data are presented as means ± 1 standard deviation (s.d.) for each dilution factor. Mean δ^15^N values of different dilution factors of the cell line were compared to the mean value of the lowest dilution factor (the highest number of cells) with the paired two-tailed Student’s test, e.g., to test for significant difference between the mean value of the highest cell number (40,000 cells, ~ 80 nmol N) and the mean value of all lower cell numbers down to 2000 cells (~ 4–5 nmol N). A *p*-value of < 0.05 was considered statistically significant.

Mouse data are presented as means ± 1 s.d. for each mouse unless indicated otherwise and were compared between groups with the paired two-tailed Student’s t-test (e.g., to test for significant difference between healthy and tumor tissue). A *p*-value of < 0.05 was considered statistically significant. In the Additional File, in SI Tables 2, 3 and 4, the δ^15^N values, standard deviation of the analyses and the number of analyses are reported for all mouse tissue samples.

## Results

### Evaluation of method performance

We evaluated the robustness of our new analytical method at low N quantities by performing a series of dilutions of breast cancer cells MCF7 and cervical cancer cells HeLa. The two cell lines analyzed showed no significant differences in mean δ^15^N between any of the different dilutions (HeLa: *n* = 14; MCF7: n = 14) and the δ^15^N of the 40,000-cell (i.e., undiluted) samples (HeLa: *n* = 6; MCF7: *n* = 4; MCF7 *p* = 0.93; HeLa *p* = 0.27). All dilution steps show good sample reproducibility for δ^15^N (Fig. 1), demonstrating reliable measurement (an accuracy relative to 40,000 cells of ±0.2‰, ±1 s.d.) of as low as 2000 cells. The precision and accuracy observed for both individual dilutions and across the broad range of dilutions indicates that the conversion of the sample N to nitrate and then N_2_O was uniformly complete, including in the samples with the greatest cell number (equivalent to ~ 80 nmol N). This finding is further supported by the high precision obtained by many replicate analyses of tissue samples with different N quantities (reported below), as well as by repeated measurements of the standard USGS65 [65] across a wide range of N quantities (10 to 800 nmols) that covers the range of N quantities of our tissue samples (Additional File, SI Table 5). Direct measurements of the analytical blank indicate an average blank size of 0.3 ± 0.1 nmol N (i.e. < 0.5% of the N quantity of the smallest measured tissue samples). The high accuracy and precision obtained for repeated analysis of international reference amino acid N standards [58, 59] within and across different batches (i.e. an accuracy with respect to the reference value of − 0.001 ± 0.06‰ for USGS40, and + 0.10 ± 0.47‰ for USGS41) indicates that our estimate of the contribution of the analytical blank is robust (Additional File, SI Tables 2, 3 and 4).

### Brain tumor ^15^N/^14^N

**Fig. 3:**
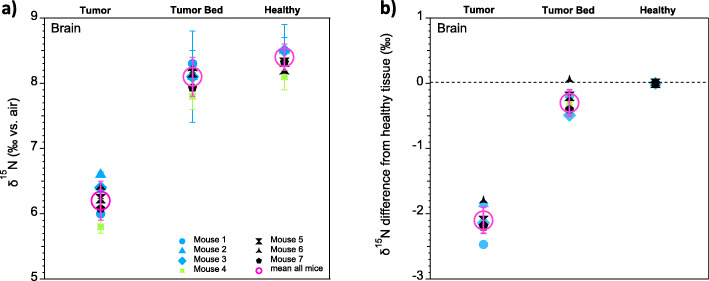
Overview of the mouse brain tumor model δ^15^N data. **a** Mean δ^15^N of all samples from the brain tumor-bearing mice (open magenta circles), plotted along with the replicate averages from individual mice. **b** For all mice, difference in δ^15^N of tumor and tumor bed from healthy tissue (the last being the reference value in **(b)** and so averaging zero by definition). In **a**, the error bars on the means of all mice indicate the standard deviations of all measurements, while the error bars on values from individual mice indicate the standard deviations of the tissue replicates. In **b**, the error bars on the means of all mice indicates the standard deviation of all measurements

The analyzed brain samples show significant differences in mean δ^15^N between the different tissue types, i.e., tumor-to-tumor bed and tumor-to-healthy tissue (*p* < 0.001; *n* = 11, where n is number of analyses per tissue type, measured across all experiments and mice). All three experiments yielded similar results (Fig. 2). Tumor tissue is characterized by lower δ^15^N than are tumor bed and healthy tissue. Individual replicates of each tissue type show coherent values with low standard deviations, indicating that the different tissue types are each homogenous with respect to their δ^15^N (Additional File SI Tables 2, 3 and 4). Across all mice analyzed, the δ^15^N difference between tumor and healthy tissue is between − 1.8‰ and − 2.5‰, yielding a multi-mouse average δ^15^N difference of − 2.1‰ ± 0.2‰ (Fig. 3).

### Head&neck tumor ^15^N/^14^N

**Fig. 4:**
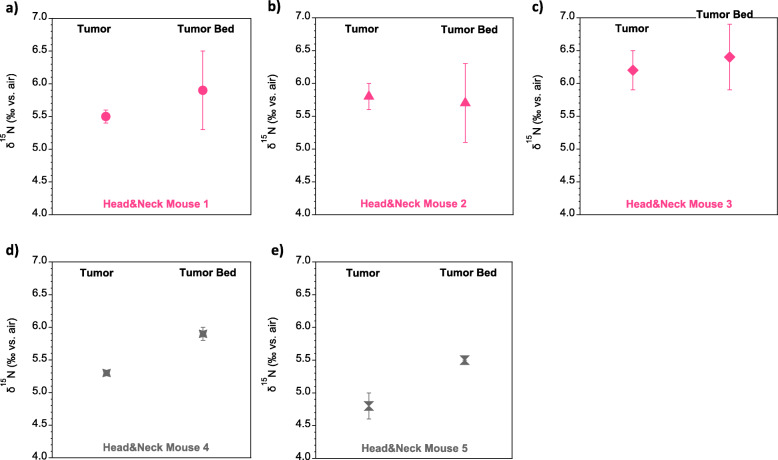
δ^15^N measurements of the mouse head&neck tumor model. **a-c** Head&neck tumors from March 2019 with 3 replicates of each tissue type (tumor, tumor bed) per mouse and 3 mice measured; **d-e** Head&neck tumors from August 2019 with 2 replicates of each tissue type (tumor, tumor bed) per mouse and  2 mice measured. Error bars indicate standard deviations of replicates

In the head&neck tumor data from March 2019 (Fig. 4 a-c), the mean difference in δ^15^N between tumor and tumor-surrounding tissue is not statistically significant (*p* = 0.4; *n* = 9, where n is number of analyses per tissue type, measured across the 3 different mice). Across the 3 mice from this first sampling effort, the variability of replicate samplings of the tumor bed was notably higher than that of the tumor (error bars indicate the normal standard deviation). This latter observation suggests a background δ^15^N difference between tumor and tumor bed, with sporadic incidental contamination of the tumor bed sample by tumor.

In the second head&neck tumor experiment from August 2019 (Fig. 4 d-e), the δ^15^N difference is more pronounced, and mean δ^15^N difference is statistically significant (*p* = 0.02; *n* = 4, where n is number of analyses per tissue type, measured across the 2 different mice). Individual replicates of tissue types (tumor, tumor bed) show smaller standard deviations than in the first experiment, consistent with more pure samplings of the tumor and/or tumor bed.

**Fig. 5:**
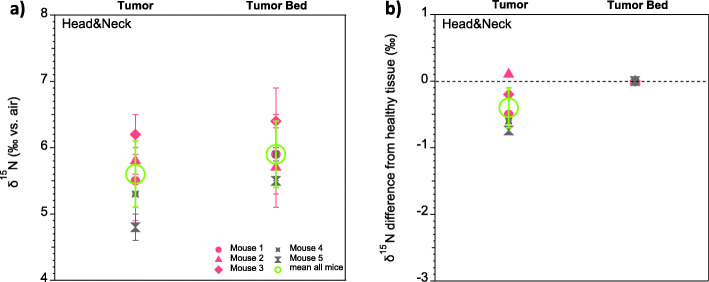
Overview of the mouse head&neck tumor model δ^15^N data. **a** Mean δ^15^N of all head&neck tumor samples (open green circles), plotted along with the replicate averages from individual mice; **b** For all mice, difference in δ^15^N of tumor from tumor bed. Error bars are as in Fig. 3

Across all head&neck tumor experiments, the total range in δ^15^N difference between tumor and tumor bed is between − 0.7‰ and 0.1‰, yielding a multi-mouse mean δ^15^N difference of − 0.4‰ (*p* = 0.1; *n* = 13, where n is number of analyses per tissue type, measured across all experiments and mice), with an average standard deviation of 0.3‰ (Fig. 5). This δ^15^N difference is much smaller than in brain cancer samplings. It remains to be resolved whether this indicates a small inherent δ^15^N difference between malignant and benign tissue associated with head&neck cancer, the intermingling of malignant and benign tissue in the head&neck sampling, or both.

## Discussion

Previous studies indicate that cancer cells, being highly proliferative, conduct more biosynthesis relative to metabolism than is the case for normal cells. Consequently, the net N-to-C ratio of their nutritional requirements is higher, which leads to their high affinity for glutamine [25, 32, 34]. Cancer cells have been shown to actively recycle metabolically produced ammonium [33], which should be depleted in ^15^N. This recycling allows cancer cells to synthesize new N-bearing compounds when other N sources (e.g., amino acids) are not provided to the cells at an adequate rate. Our data support the hypothesis that reincorporation of this low-^15^N metabolic ammonium in cancer cells results in a lower δ^15^N of tumor tissue relative to the surrounding heathy tissue, in which low-^15^N metabolic N is not recycled but rather released to the extracellular environment. If so, healthy cells are enriched in ^15^N with respect to their N source [36], while cancer cells have a δ^15^N more similar to that of their N source. The observation that glutamate dehydrogenase is overexpressed in various cancer types [33] supports the possibility that the δ^15^N difference between cancer and healthy tissues reported here is a general feature, both in rodents and humans.

To our knowledge, five studies have reported natural abundance δ^15^N measurements of cancer biopsies [14–18]. Taran et al. 2015 [14] measured the δ^15^N and δ^13^C (δ^13^C in permil, ‰ = ([(^13^C/^12^C)_sample_/(^13^C/^12^C)_PDB_]-1)*1000) of tissue biopsies of 13 animal muscle samples, in order to establish the optimal size for measurement. They then proceeded to study the δ^15^N and δ^13^C of human tumor tissue samples. The measurements were done by automated Dumas combustion, which converts the C and N in the sample to CO_2_ and N_2_, under a continuous helium flow (using an instrument often referred to as an elemental analyzer (EA)) that is plumbed to an isotope ratio mass spectrometer (IRMS). This technology is known as EA-CF-IRMS (CF- for continuous flow) [66]. EA-CF-IRMS instrumentation is widely available, but off-the-shelf technology requires relatively large sample quantities to produce accurate measurements (≥1–2 μmol N in the most sensitive systems, > 200 higher than the ~ 5 nmol N needed in our analyses). Taran et al. estimated that 5 mg of tissue per sample was ideal for the analysis, but indicated that measurements were feasible down to 0.5 mg. Samples from fine needle aspiration biopsies were found to be insufficient to obtain accurate measurements [14]. In another study, Taran et al. 2015 [16] compared the δ^13^C and δ^15^N of 6 embryonal and 2 alveolar rhabdomyosarcoma biopsies (~ 5 mg tissue per sample) from 8 different patients [16]. They observed an elevation in the mean δ^15^N of patients with embryonal rhabdomyosarcoma (9.15‰ ± 1.32‰) with respect to those with the alveolar type (8.01‰ ± 1.12 ‰). No comparison with healthy tissue was done. Taran et al. 2016 [15] measured the δ^15^N of 84 Wilms tumor tissue samples from different nephroblastoma histological types at different stages of this disease. Subsequent stages of the disease varied between 8.11‰ ± 2.73‰ to 8.66‰ ± 0.51‰, with the lowest δ^15^N in stage 3 of the disease. Two samples of normal kidney tissues were measured (samples obtained from two car accident victims from the same region as the cancer patients), yielding a mean δ^15^N of 7.61‰ ± 0.18‰.

None of the above studies compared the δ^15^N of the corresponding tumor and healthy tissue from the same patients. This limitation was inherent to working with samples from human patients, as there was not a medical justification for sampling of healthy tissue. Going forward, in the diagnostic setting, the ability to compare suspected malignant and corresponding healthy tissue from a given organ would be greatly aided by the higher sensitivity methods that are employed in the present study, which would allow for fine scale sampling of micro-biopsies.

Bogusiak et al. [18] compared the δ^13^C and δ^15^N of oral squamous cell carcinoma to margin and healthy tissue in 18 patients. Two tumor, two margin and two healthy oral mucosa samples per patient were measured. Samples of 3 mg ± 1 mg were analyzed by EA-IRMS; needle aspiration biopsy was found to yield inadequate sample size. The mean δ^15^N value of the 18 patients combined was lower in tumor than in healthy tissue (8.92‰ ± 0.58‰ and 9.84‰ ± 0.61‰, respectively), consistent with our murine results. However, a δ^15^N comparison of the corresponding tumor and healthy tissue within the same patients was not reported.

Tea et al. [17] used the same methods to measure the δ^13^C and δ^15^N of 5 pairs of breast cancer and adjacent non-cancerous tissues of relatively large biopsies (several mg of tissue per sample) from chemotherapy-naive human patients. The results of these analyses show a statistically significant difference in the δ^13^C between tumor samples and adjacent healthy tissue. While the authors perceived a lower δ^15^N in the cancerous tissue, the δ^15^N difference from healthy tissue was not statistically significant.

The general relevance of N isotopic signature for cancer is supported by two studies of the δ^15^N of cancer cells in vitro [17, 24]. Tea et al. compared the δ^15^N of a non-cancerous immortalized mammary epithelial cell line (MCF10A) with six breast cancer cell lines (ZR75–1, MCF7, SKBR3, MDA-MB-231, MDA-MB-468 and CAL51) grown in vitro, but using different growth conditions for the cancerous and healthy cell lines. Their results show that, with the exception of ZR75–1, all breast cancer cell lines were lower in δ^15^N by ~ 1 to 3‰ with respect to the non-cancerous line [17]. Krishnamurthy et al. compared the δ^15^N of four human colorectal cancer cell lines (SW1463, WiDr, HCT 116 and COLO 205) with those of one fetal human fetal lung fibroblast cell line (WI38) grown in vitro [24]. Their results showed that colorectal cancer cell lines were lower in δ^15^N by ~ 4 to 5‰ relative to the healthy fetal lung fibroblasts, and they identified glutamic acid as being the main driver for the observed differences. Although the results of these two in vitro studies are encouraging, the use of different growth media for cancerous and non-cancerous cell types in the first study and the use of non-analogous cell types for cancerous vs. non-cancerous cells in the second study hamper the attribution of the observed isotopic differences. The δ^15^N differences may have derived from different metabolisms associated with carcinogenesis, different metabolisms associated with cell and tissue type, or different availabilities of N sources (with distinct δ^15^N values) among media formulations.

Our study was performed using two tumor types of different tissue origin: GBM, which is a glial tumor of the brain, and head&neck carcinoma, which is of epithelial origin. In both cases, we found a lower δ^15^N in tumor tissue, suggesting that this could be a general feature of tumors. However, the δ^15^N difference between tumor and healthy tissue was smaller for the head&neck cancer model (− 0.4‰ ± 0.3‰) than for the brain cancer model (− 2.1‰ ± 0.2‰). Challenges with separating malignant and healthy tissue in the head&neck cancer model likely contributed to this observation. Thus, mixing of the two tissue end-members, cancerous and healthy, through infiltration or other processes, will likely prove to influence the use of micro-biopsy N isotopic analysis in some cancer types. The relatively small standard deviations associated with the replicates of the second sampling effort of head&neck cancer tissues (Fig. 4 d, e) argue for a more robust separation of tissues than in the first sampling effort. Yet, in this second experiment, the measured tumor-tumor bed δ^15^N difference was still small, roughly − 0.7‰. This suggests that the tumor-healthy tissue δ^15^N difference is indeed smaller in the head&neck cancer model than in the brain cancer model.

**Fig. 6:**
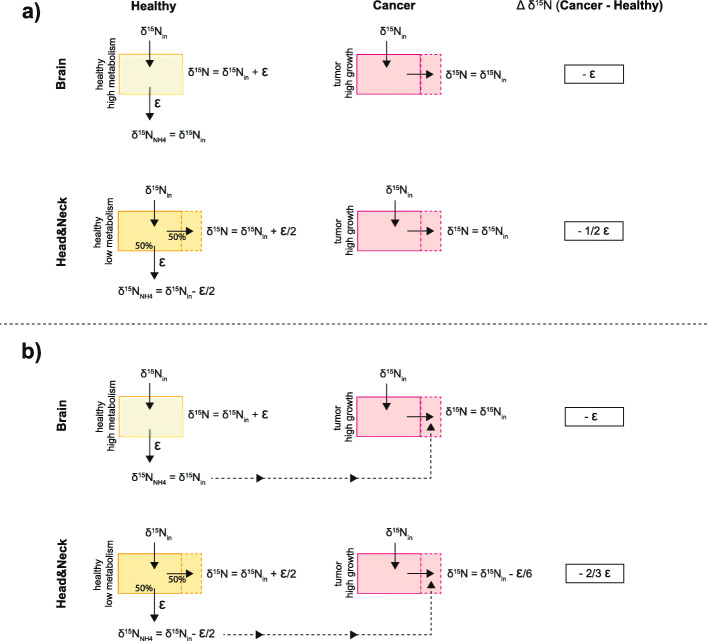
Illustration of the impacts of different ratios of metabolic N efflux and growth on tissue δ^15^N, as an explanation for the lower δ^15^N of tumor tissue. δ^15^N_in_ is the δ^15^N of external N supply to the organ in question. ε is a measure of the amplitude of the isotope effect of ammonium catabolism and efflux. Here, a higher ε denotes a stronger preference for ^14^N relative to ^15^N in the conversion (see text). Δδ^15^N(cancer-healthy) refers to the δ^15^N difference between tumor tissue and healthy equivalent. Box colors are as follows: green: healthy tissue with high metabolism (100% of N input is effluxed as ammonium); yellow: healthy tissue with low metabolism (or more precisely, low metabolism-to-growth ratio, with 50% of N input effluxed as ammonium); red: tumor tissue with high growth and no ammonium efflux. In (**a**), healthy and tumor tissues are considered as isolated. In (**b**), as part of its N supply, tumor tissue also taps extracellular ammonium effluxed from healthy tissue. Tops of both (**a**) and (**b**): high metabolism mode (qualitatively corresponding to neural tissue in our experiments). Bottoms of (**a**) and (**b**): low metabolism mode (corresponding to head&neck tissue)

Murine studies indicate that neural tissue has a high δ^15^N in comparison to the rest of the organism [19]. Our data are consistent with this result, in that we observed a higher δ^15^N in healthy neural tissue than in healthy head&neck (muscular, skin, and salivary gland) tissues, while both mice models were grown in the same lab and on the same diet. δ^15^N differences among tissues are believed to be related to N metabolic activity [21], and this likely results from the release of low-δ^15^N metabolic ammonium, with higher ammonium loss resulting in a higher tissue δ^15^N (Fig. 6). The preference for ^14^N and against ^15^N in such a reaction is often quantified as the ε (or “epsilon value”) of the reaction. ε is defined here as (1-^15^k/^14^k), where ^14^k and ^15^k refer to the ^14^N- and ^15^N-associated rate coefficients for the reaction; it is put in units of permil (‰, per thousand) by multiplying by 1000. Here, the reaction of interest is the catabolic production of ammonium, which leads to its efflux from the tissue. ε is well approximated as the δ^15^N difference between the substrate (here, the tissue N) and the product being produced instantaneously from it (here, the ammonium being produced catabolically from the tissue and released into the extracellular environment). Physiological and ecological studies suggest a tissue-scale value for ε of 3–4‰ [19, 20, 36–38, 40].

The greater the fraction of assimilated N that is routed to metabolic ammonium loss as opposed to growth, the higher the δ^15^N of the tissue should be. This is illustrated in Fig. 6 considering two alternative scenarios. In Fig. 6 a, it is assumed that malignant and healthy tissues of a given organ are isolated from each other. In Fig. 6 b, it is assumed that the metabolic ammonium flux from the healthy tissue serves as an additional N source to malignant cells. In both scenarios, we distinguish a healthy tissue with high metabolism (“healthy high metabolism”: 100% of N supply ultimately resulting in catabolic ammonium efflux; green rectangles in Fig. 6) and a healthy tissue with a lower metabolism-to-growth ratio (“healthy low metabolism”: 50% of N supply going to ammonium efflux; yellow rectangles in Fig. 6). In the case of high metabolic activity in a healthy tissue (e.g., brain), most of the N supply to the tissue is catabolized to ammonium. The resulting δ^15^N in the tissue is therefore driven higher by the isotope effect of catabolic deamination and leads to tissue δ^15^N elevation compared to its source (δ^15^N = δ^15^N_in_ + ε). In the case of lower metabolic activity (e.g., head&neck tissue), N input is partially routed to growth and less ammonium loss occurs (assuming equal proportions of each in our hypothetical scenario). In this case, the δ^15^N of tissue tends to be less elevated relative to its N inputs than in the case of high metabolic activity (δ^15^N = δ^15^N_in_ + ε/2).

Due to cancer cells’ high metabolic needs, it is expected and observed that cancer cells assimilate ammonium [7], suggesting that they allow little metabolic ammonium to escape to the bloodstream. In Fig. 6, for the sake of a quantitatively simple argument, we assume that cancer cells represent the end-member case of no net catabolic ammonium loss, with all N input being put toward growth (“tumor high growth,” pink rectangles). For the isolated tissue scenario of Fig. 6 a, tumor tissue will have the δ^15^N of the N supplied to it (δ^15^N = δ^15^N_in_). This results in lower δ^15^N in the tumor tissue than in corresponding healthy tissue: in Fig. 6, “Δδ^15^N(cancer-healthy)” is negative.

The lowering of tumor tissue δ^15^N relative to corresponding healthy tissue is strongest in the case of an organ with a high metabolic rate; that is, in an organ with a greater metabolic rate, Δδ^15^N(cancer-healthy) is of greater amplitude. However, the magnitude of Δδ^15^N(cancer-healthy) is different in the two scenarios proposed in Fig. 6 a and 6 b. In Fig. 6 a, Δδ^15^N(cancer-healthy) = − ε/2 in the organ with a low metabolism vs. Δδ^15^N(cancer-healthy) = − ε in an organ with high healthy metabolism. In Fig. 6 b, in which the metabolic ammonium flux from the healthy tissue serves as an additional N source to malignant cells, the contrast in Δδ^15^N(cancer-healthy) between the two tissue types is weaker (− 2/3ε vs. -ε; Fig. 6 b). Nevertheless, the distinction in Δδ^15^N(cancer-healthy) between high-metabolism and low-metabolism organs still exists.

The simple calculations of Fig. 6 are for demonstrative purposes only, and the values used (e.g., for the proportion of N routed to growth vs. metabolism) are purely hypothetical. A quantitative and conclusive analysis will require more data and will avail itself of other physiological information. With regard to isotope dynamics, in the case of ammonium transfer from healthy to malignant cells (Fig. 6 b), the potential for isotopic discrimination during the ammonium assimilation should be considered [67]. Nevertheless, the simple calculations conducted here illustrate that the particularly strong δ^15^N lowering in the brain cancer samples relative to healthy neural tissue may be due to the cancer’s canceling of very high metabolic ammonium release that typifies healthy neural tissue. In contrast, the weaker δ^15^N lowering in the head&neck cancer samples relative to healthy head&neck tissue might reflect the more limited metabolic ammonium release from muscle and structural tissues.

If so, there are implications for human cancers. For example, unlike mice, adult humans have ceased net growth. As a result, different human tissues should tend to have less divergent δ^15^N values and, overall, a higher δ^15^N relative to N sources. In this case, Δδ^15^N(cancer-healthy) should tend to be more robust and less variable across tumor types in humans. This can be tested in future work.

Alternatively, it may simply be a coincidence that the higher cancer-healthy tissue δ^15^N difference occurred in an organ (the brain) with a high δ^15^N. If so, the characteristics of the tumor itself (e.g., its avidity for metabolically generated ammonium) may be the dominant control on the amplitude of the δ^15^N difference between malignant and healthy tissue. Pursuing these different possible explanations (e.g., by conducting a similar study with other cancer types) promises to provide further insight into the acquisition and conservation of N by tumor cells compared to healthy cells.

## Conclusions

Using novel nitrogen isotopic analysis methods, we have undertaken an initial comparative study of cancerous and healthy tissues in two cancer mice models. The results indicate a robust nitrogen isotopic difference between malignant and healthy tissue within a given organ and a given individual, offering support for its diagnostic application. In addition, the nitrogen isotopic differences between tissues might help to quantify the level of tumor cell infiltration at tumor margin, which is one of the most challenging issues after surgery as it affects tumor recurrence. Much more work, in both non-human models and in cancer patients, is needed to evaluate the potential of the natural abundance nitrogen isotope ratios to yield insights into cancer metabolism and provide new diagnostic avenues.

The high-sensitivity methods employed here will greatly facilitate this exploration. The higher sensitivity of our analytical method compared to previous studies allows us to better target the end-members of tumor, tumor microenvironment and surrounding healthy tissue. This can contribute to reducing the need for invasive procedures because it is suitable for samples from fine needle aspiration biopsies. The method is able to provide results in a relatively short timeframe (48 h or less) and might therefore support histopathology in cancer diagnosis. In the research setting, detailed analyses of micro-biopsies could be undertaken, in order to better understand the nitrogen flows between cancer cells and surrounding tissues.

## Supplementary Information

**Additional file 1: Figure SI-1**. Illustration of a tumor mass formed by H454 tumors (T) in the striatum of the neural and adjacent tissue (TB = tumor bed). The healthy, normal neural tissue (NT) was sampled in the contralateral region, which is tumor-free. **Figure SI-2**. The left panel shows an illustration of the tumor mass formed by MEERL95 tumors (T = tumor) in the submandibular region. It infiltrates the adjacent healthy subcutaneous and muscular tissues (S=skin, SG = salivary gland, M = muscle). The right panel shows a 20-fold higher magnification of the margin (m). Infiltration of tumor cells (purple) into the stromal tissue (pink) forms an irregular margin (m). Intravascular tumor cell aggregates and smaller clusters of tumor cells are also found further away from the primary tumor in the subcutaneous zone (*). For our study, margin and tumor were sampled. **Table SI-1**. Isotopic measurements performed on the cell samples. At the bottom of the table,  average values for reference standards and procedural oxidation blank are shown. **Table SI-2**. Isotopic measurements performed in April 2019. The data includes the Head&Neck data from mice 1, 2 and 3 and the brain tumor data from mice 1, 2 and 3. At the bottom of the table, values for reference standards and procedural oxidation blank are shown. **Table SI-3**. Isotopic measurements performed in September 2019. The data include the Head&Neck data from mice 4 and 5. At the bottom of the table values for reference standards and procedural oxidation blank are shown. **Table SI-4**. Isotopic measurements performed in November 2019. The data includes the brain tumor data from mice 4, 5, 6 and 7. At the bottom of the table, values for reference standards and procedural oxidation blank are shown. **Table SI-5**. Results of USGS65 measurements across the range of N contents of our tissue samples. Our results show no statistically significant difference in δ^15^N, indicating full conversion of the organic N during the oxidation step.

## Data Availability

The datasets generated and analyzed during the current study are available in the Additional file 1.

## Abbreviations

N: Nitrogen
C: Carbon
HPW: High purity water
USGS: U.S. geological survey
IAEA: International atomic energy agency
DMEM: Dulbecco’s modified eagle’s medium
FBS: Fetal bovine serum
PBS: Phosphate buffered saline
s.d.: Standard deviation
GBM: Glioblastoma
EA: Elemental analyzer
CF: Continuous flow
IRMS: Isotope radio mass spectrometer
EA: Elemental analyzer
