# Supplementary material for: Distinct nitrogen isotopic compositions of healthy and cancerous tissue in mice brain and head&neck micro-biopsies
Source: BMC Cancer. 2021 Jul 13;21:805. doi: 10.1186/s12885-021-08489-x (PMC8276491; doi:10.1186/s12885-021-08489-x)
Supplement: Supplementary file 1 — Additional file 1: Figure SI-1. Illustration of a tumor mass formed by H454 tumors (T) in the striatum of the neural and adjacent tissue (TB = tumor bed). The healthy, normal neural tissue (NT) was sampled in the contralateral region, which is tumor-free. Figure SI-2. The left panel shows an illustration of the tumor mass formed by MEERL95 tumors (T = tumor) in the submandibular region. It infiltrates the adjacent healthy subcutaneous and muscular tissues (S=skin, SG = salivary gland, M = muscle). The right panel shows a 20-fold higher magnification of the margin (m). Infiltration of tumor cells (purple) into the stromal tissue (pink) forms an irregular margin (m). Intravascular tumor cell aggregates and smaller clusters of tumor cells are also found further away from the primary tumor in the subcutaneous zone (*). For our study, margin and tumor were sampled. Table SI-1. Isotopic measurements performed on the cell samples. At the bottom of the table, average values for reference standards and procedural oxidation blank are shown. Table SI-2. Isotopic measurements performed in April 2019. The data includes the Head&Neck data from mice 1, 2 and 3 and the brain tumor data from mice 1, 2 and 3. At the bottom of the table, values for reference standards and procedural oxidation blank are shown. Table SI-3. Isotopic measurements performed in September 2019. The data include the Head&Neck data from mice 4 and 5. At the bottom of the table values for reference standards and procedural oxidation blank are shown. Table SI-4. Isotopic measurements performed in November 2019. The data includes the brain tumor data from mice 4, 5, 6 and 7. At the bottom of the table, values for reference standards and procedural oxidation blank are shown. Table SI-5. Results of USGS65 measurements across the range of N contents of our tissue samples. Our results show no statistically significant difference in δ15N, indicating full conversion of the organic N during the oxidation step. [file 12885_2021_8489_MOESM1_ESM.pdf]

Additional File 1

M. Straub[1;2], D.M. Sigman[3], A. Auderset[2], J. Ollivier[4], B. Petit[4], B. Hinnenberg[2], F. Rubach[2], S. Oleynik[3], M.-C. Vozenin[4,] A. Martínez-García[2]

[1] Institute of Radiation Physics, Lausanne University Hospital and University of Lausanne, 1007 Lausanne, Switzerland
[2] Max Planck Institute for Chemistry, 55128 Mainz, Germany
[3] Department of Geosciences, Princeton University, Princeton, NJ 08544
[4] Radiation Oncology Laboratory/DO/Radio-Oncology/CHUV, Lausanne University Hospital and University of Lausanne, 1011 Lausanne, Switzerland

Illustrations of histopathological samples

For SI Figure 1, H454 tumors, whole brain was sampled, cryoprotected (30% sucrose) and sectioned coronally (30 – 35 µm thick) using a cryostat (Leica Microsystems, Germany). Sections were imaged after HE staining by histopathology. For SI Figure 2, MEERL95, tumors were sampled and 4 µm formalin-fixed paraffin-embedded (FFPE) sections were HE stained and used for histopathological analysis. Example images of the two tumor types are from different mice than used in this study.

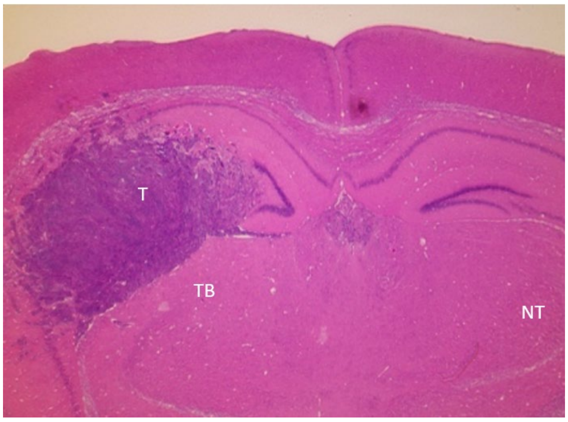

SI Figure 1: Illustration of a tumor mass formed by H454 tumors (T) in the striatum of the neural and adjacent tissue (TB= Tumor bed). The healthy, normal neural tissue (NT) was sampled in the contralateral region, which is tumor-free.

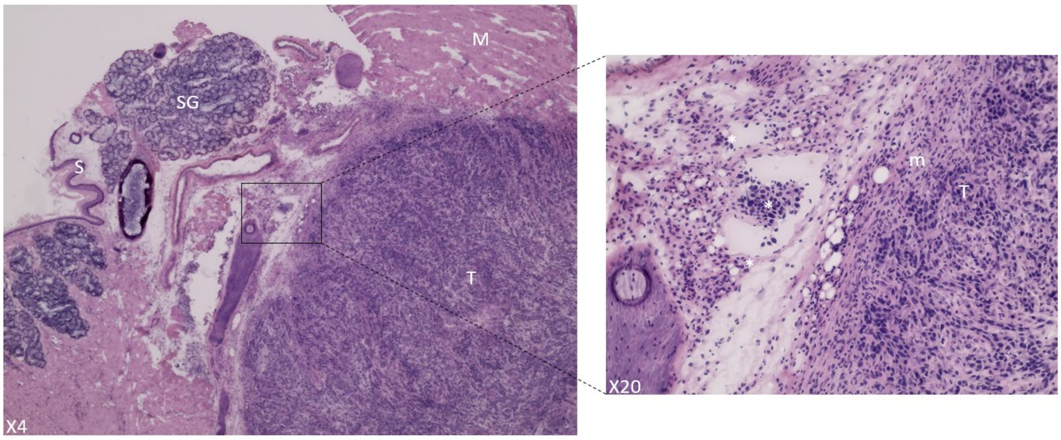

SI Figure 2: The left panel shows an illustration of the tumor mass formed by MEERL95 tumors (T=tumor) in the submandibular region. It infiltrates the adjacent healthy subcutaneous and muscular tissues (S=skin, SG= salivary gland, M=muscle). The right panel shows a 20-fold higher magnification of the margin (m). Infiltration of tumor cells (purple) into the stromal tissue (pink) forms an irregular margin (m). Intravascular tumor cell aggregates and smaller clusters of tumor cells are also found further away from the primary tumor in the subcutaneous zone (*). For our study, margin and tumor were sampled.

Experimental data from cell and tissue measurements

Experimental data for cell and tissue samples and reference standards is summarized in SI Tables 1 to 4. In all experiments, aliquots of 5nmol N quantity were analyzed. Table 5 shows the results of the USGS65 series.

SI Table 1: Isotopic measurements performed on the cell samples. At the bottom of the table, values for average reference standards and procedural oxidation blank are shown.

**MCF7**				
**Number of cells**	**Number of subsamples measured**	**Average $\delta^{15}N$**	**1 s.d.**	**Absolute difference to 40 000 cell sample**
2000	3	-1.74	0.05	-0.18
4000	2	-1.49	0.12	0.07
8000	3	-1.50	0.06	0.06
12000	2	-1.43	0.08	0.13
20000	4	-1.55	0.06	0.01
40000	4	-1.56	0.07	0.00
**HeLa**				
**Number of cells**	**Number of subsamples measured**	**Average $\delta^{15}N$**	**1 s.d.**	**Absolute difference to 40 000 cell sample**
2000	3	-1.57	0.27	-0.15
4000	2	-1.43	0.06	-0.01
8000	3	-1.44	0.04	-0.02
12000	2	-1.38	0.00	0.05
20000	4	-1.48	0.08	-0.05
40000	6	-1.42	0.04	0.00
**Average reference standards and blanks**		**N quantity per sample (nmol)**	**Mean $\delta^{15}N$**	**$\delta^{15}N$ 1 s.d.**
**Procedural Oxidation Blank***		0.39	-3.50	0.88
**USGS40 (reference value –4.5 ± 0.1 ‰)°**		5-30	-4.59	0.03
**USGS41 (reference value +47.6 ± 0.2 ‰)°°**		5-30	47.64	0.02

** Cell samples were measured in two runs. For each run measured, 8 vials with 1 ml Persulfate, merged together for 2 measurements.*
° https://isotopes.usgs.gov/lab/referencematerials/USGS40.pdf
°° https://isotopes.usgs.gov/lab/referencematerials/USGS41.pdf

SI Table 2: Isotopic measurements performed in April 2019. The data includes the Head&Neck data from mice 1, 2 and 3, and the brain tumor data from mice 1, 2 and 3. At the bottom of the table values for reference standards and procedural oxidation blank are shown.

Isotopic Measurements April 2019					
**Identification**	**Tissue Type**	**N quantity per sample (nmol)**	**$\delta^{15}N$**	**Mean $\delta^{15}N$**	**1 s.d.**
Mouse 1 H&N	Tumor	162	5.6	5.5	0.1
Mouse 1 H&N	Tumor	136	5.5		
Mouse 1 H&N	Tumor	88	5.3		
Mouse 1 H&N	Tumor Bed	353	5.7	5.9	0.6
Mouse 1 H&N	Tumor Bed	83	5.5		
Mouse 1 H&N	Tumor Bed	262	6.6		
Mouse 1 H&N	Tumor	508	6.0	5.8	0.2
Mouse 2 H&N	Tumor	243	5.6		
Mouse 2 H&N	Tumor	246	5.7		
Mouse 2 H&N	Tumor Bed	141	6.3	5.7	0.6
Mouse 2 H&N	Tumor Bed	68	5.2		
Mouse 2 H&N	Tumor Bed	99	5.6		
Mouse 3 H&N	Tumor	194	5.9	6.2	0.3
Mouse 3 H&N	Tumor	191	6.3		
Mouse 3 H&N	Tumor	214	6.4		
Mouse 3 H&N	Tumor Bed	450	6.8	6.4	0.5
Mouse 3 H&N	Tumor Bed	327	6.5		
Mouse 3 H&N	Tumor Bed	197	5.9		
Mouse 1 Brain	Tumor	153	6.2	6.0	0.2
Mouse 1 Brain	Tumor	454	5.9		
Mouse 1 Brain	Tumor Bed	266	8.4	8.3	0.2
Mouse 1 Brain	Tumor Bed	282	8.2		
Mouse 1 Brain	Healthy Tissue	183	8.4	8.5	0.2
Mouse 1 Brain	Healthy Tissue	235	8.6		
Mouse 2 Brain	Tumor	268	6.7	6.6	0.0
Mouse 2 Brain	Tumor	265	6.6		
Mouse 2 Brain	Tumor Bed	108	7.6	8.1	0.7
Mouse 2 Brain	Tumor Bed	111	8.6		
Mouse 2 Brain	Healthy Tissue	112	8.4	8.5	0.2
Mouse 2 Brain	Healthy Tissue	474	8.6		
Mouse 3 Brain	Tumor	327	6.3	6.4	0.1
Mouse 3 Brain	Tumor	503	6.5		
Mouse 3 Brain	Tumor Bed	135	7.8	8.1	0.3
Mouse 3 Brain	Tumor Bed	168	8.3		
Mouse 3 Brain	Healthy Tissue	286	8.8	8.5	0.4
Mouse 3 Brain	Healthy Tissue	77	8.2		
USGS40 5nmol	NA	5	NA	NA	NA
USGS40 15nmol	NA	15	NA		
USGS40 30nmol	NA	30	NA		
USGS41 5nmol	NA	5	NA	47.4	0.01
USGS41 15nmol	NA	15	47.4		
USGS41 30nmol	NA	30	47.4		
**Procedural Oxidation Blank***	NA	0.3	--	-10.4	NA
**Average USGS40** (reference value –4.5 ± 0.1 ‰)°**	NA	5-30	--	NA	NA
**Average USGS41** (reference value +47.6 ± 0.2 ‰)°°**	NA	5-30	--	47.4	0.01

** 4 blank vials with 1 ml Persulfate were merged together for one isotopic measurement*

*** 4 of the vials of the amino acid standards broke when opening after autoclaving, only two values of the USGS41 could be used in this run.*

° https://isotopes.usgs.gov/lab/referencematerials/USGS40.pdf

°° https://isotopes.usgs.gov/lab/referencematerials/USGS41.pdf

SI Table 3: Isotopic measurements performed in September 2019. The data includes the Head&Neck data from mice 4 and 5. At the bottom of the table values for reference standards and procedural oxidation blank are shown.

Isotopic Measurements September 2019					
**Identification**	**Tissue Type**	**N quantity per sample (nmol)**	**$\delta^{15}N$**	**Mean $\delta^{15}N$**	**1 s.d.**
Mouse 4 H&N	Tumor	141	5.3	5.3	0.0
Mouse 4 H&N	Tumor	277	5.3		
Mouse 4 H&N	Tumor Bed	171	5.9	5.9	0.1
Mouse 4 H&N	Tumor Bed	180	5.8		
Mouse 5 H&N	Tumor	259	4.6	4.8	0.2
Mouse 5 H&N	Tumor	173	4.9		
Mouse 5 H&N	Tumor Bed	391	5.5	5.5	0.0
Mouse 5 H&N	Tumor Bed	204	5.5		
USGS40 5nmol	NA	5	-4.6	-4.5	0.1
USGS40 15nmol	NA	15	-4.5		
USGS40 30nmol	NA	30	-4.5		
USGS41 5nmol	NA	5	46.4	47.3	0.8
USGS41 15nmol	NA	15	47.7		
USGS41 30nmol	NA	30	47.8		
Procedural Oxidation Blank*	NA	0.5	--	1.0	0.1
Average USGS40** (reference value –4.5 ± 0.1 ‰)°	NA	5-30	--	-4.5	0.1
Average USGS41** (reference value +47.6 ± 0.2 ‰)°°	NA	5-30	--	47.3	0.8

** 10 vials with 1 ml Persulfate, merged together for 2 measurements*
° https://isotopes.usgs.gov/lab/referencematerials/USGS40.pdf
°° https://isotopes.usgs.gov/lab/referencematerials/USGS41.pdf

SI Table 4: Isotopic measurements performed in November 2019. The data includes the brain tumor data from mice 4, 5, 6 and 7. At the bottom of the table, values for reference standards and procedural oxidation blank are shown.

Isotopic measurment November 2019					
**Identification**	**Tissue Type**	**N quantity per sample (nmol)**	**$\delta^{15}N$**	**Mean $\delta^{15}N$**	**1 s.d.**
Mouse 4 Brain	Tumor	164	5.8	5.8	0.1
Mouse 4 Brain	Tumor	253	5.9		
Mouse 4 Brain	Tumor Bed	97	7.7	7.8	0.2
Mouse 4 Brain	Tumor Bed	101	7.9		
Mouse 4 Brain	Healthy Tissue	135	7.9	8.1	0.2
Mouse 4 Brain	Healthy Tissue	124	8.2		
Mouse 5 Brain	Tumor	199	6.2	NA	NA
Mouse 5 Brain	Tumor Bed	109	8.2	NA	NA
Mouse 5 Brain	Healthy Tissue	103	8.3	NA	NA
Mouse 6 Brain	Tumor	129	6.4	NA	NA
Mouse 6 Brain	Tumor Bed	106	8.2	NA	NA
Mouse 6 Brain	Healthy Tissue	116	8.2	NA	NA
Mouse 7 Brain	Tumor	199	6.1	NA	NA
Mouse 7 Brain	Tumor Bed	97	7.9	NA	NA
Mouse 7 Brain	Healthy Tissue	92	8.3	NA	NA
USGS40 5nmol	NA	5	-4.5	-4.5	0.1
USGS40 15nmol	NA	15	-4.6		
USGS40 30nmol	NA	30	-4.5		
USGS41 5nmol	NA	5	47.6	47.7	0.1
USGS41 15nmol	NA	15	47.7		
USGS41 30nmol	NA	30	47.8		
Procedural Oxidation Blank*	NA	0.2	--	-2.3	0.1
Average USGS40** (reference value –4.5 ± 0.1 ‰)°	NA	5-30	--	-4.5	0.1
Average USGS41** (reference value +47.6 ± 0.2 ‰)°°	NA	5-30	--	47.7	0.1

** 10 vials with 1 ml Persulfate, merged together for 2 measurements*
° https://isotopes.usgs.gov/lab/referencematerials/USGS40.pdf
°° https://isotopes.usgs.gov/lab/referencematerials/USGS41.pdf

SI Table 5: Results of USGS65 measurements across the range of N contents of our tissue samples. Our results show no statistically significant difference in $\delta^{15}N$ indicating a full conversion of the organic N during the oxidation step.

**Sample Name**	**$\delta^{15}N$**
USGS65 10 nmol of N	20.63
USGS65 10 nmol of N	20.75
USGS65 50 nmol of N	20.57
USGS65 50 nmol of N	20.55
USGS65 100 nmol of N	20.47
USGS65 100 nmol of N	20.34
USGS65 100 nmol of N	20.41
USGS65 400 nmol of N	20.62
USGS65 400 nmol of N	20.64
USGS65 400 nmol of N	20.53
USGS65 800 nmol of N	21.21
USGS65 800 nmol of N	20.88
USGS65 800 nmol of N	20.88
**Average $\delta^{15}N$**	**20.7**
**1 s.d.**	**0.2**
**Reference value USGS65°**	**+20.68 ± 0.06 ‰**

°https://isotopes.usgs.gov/lab/referencematerials/USGS64-USGS65-USGS66.pdf
